# Corporate headquarters in the twenty-first century: an organization design perspective

**DOI:** 10.1186/s41469-020-00086-9

**Published:** 2020-11-05

**Authors:** Sven Kunisch, Markus Menz, David Collis

**Affiliations:** 1 Department of Business Development and Technology, Aarhus University, Birk Centerpark 15, 7400 Herning, Denmark; 2 Geneva School of Economics and Management, University of Geneva, Boulevard du Pont D’Arve 40, 1211 Geneva, Switzerland; 3 Harvard Business School, Harvard University, Soldiers Field, Boston, MA 02163 USA

**Keywords:** Corporate headquarters, Corporate center, Organization design, Theory of the firm, Strategy, International business, Organization theory, Digital age, Sustainability

## Abstract

The corporate headquarters (CHQ) of the multi-business enterprise, which emerged as the dominant organizational form for the conduct of business in the twentieth century, has attracted considerable scholarly attention. As the business environment undergoes a fundamental transition in the twenty-first century, we believe that understanding the evolving role of the CHQ from an organization design perspective will offer unique insights into the nature of business activity in the future. The purpose of this article, in keeping with the theme of the *Journal of Organization Design* Special Collection, is thus to invigorate research into the CHQ. We begin by explicating four canonical questions related to the design of the CHQ. We then survey fundamental changes in the business environment occurring in the twenty-first century, and discuss their potential implications for CHQ design. When suitable here we also refer to the contributions published in our Special Collection. Finally, we put forward recommendations for advancements and new directions for future research to foster a deeper and broader understanding of the topic. We believe that we are on the cusp of a change in the CHQ as radical as that which saw its initial emergence in the late nineteenth/early twentieth century. Exactly what form that change will take remains for practitioners and researchers to inform.

## Introduction

The corporate headquarters (CHQ) is a key feature of diversified business organizations that have dominated the economies of developed countries for over a century (Chandler 1962, 1991; Menz et al. 2015). It was Chandler’s (1962) seminal work on the Industrial Enterprise in the twentieth century that fueled scholarly attention on the “modern” CHQ as a separate organizational entity in multi-business firms. Chandler described the emergence of the multidivisional (M-form) organization with a CHQ in which “general executives and staff specialists coordinate, appraise, and plan goals and policies and allocate resources for a number of quasi-autonomous, fairly self-contained divisions” (Chandler 1962, p. 9), which was later acknowledged to be probably the most noteworthy organizational innovation of the twentieth century (Chandler 1991, 1992; Williamson 1985).

It therefore comes as little surprise that scholars in various fields including strategy (Chandler 1991; Rumelt et al. 1994), organization studies (Mintzberg 1979), organizational economics (Foss 1997, 2019), as well as corporate finance (Bolton and Scharfstein 1998; Scharfstein 1998) and international business (IB) (Buckley and Casson 2020; da Silva Lopes et al. 2018; Hedlund 1984) have examined the nature and functioning of the CHQ (see Table 1).^1^ While the research foci, theories, and methodologies vary across these streams, their collective efforts have contributed to a comprehensive understanding of the CHQ and elucidated insights into the functioning of the “modern” business organization in the industrial age during the twentieth century.

**Table 1:** Research on CHQ design in various fields

	Strategic management^a^	Organizational economics	International business	Organization studies	Corporate finance
General concerns	Corporate advantage Resource allocation Organization Structure	Scope and boundaries of the firm Transactions costs Agency and incentives Governance	International advantage Location Operating unit/CHQ relationships	Differentiation–integration Authority Information-processing	Corporate governance Corporate diversification Resource allocation
Foundational studies	Chandler (1962) Bower (1970) Goold and Campbell (1987)	Coase (1937) Williamson (1975, 1985)	Perlmutter (1969) Stopford and Wells Jr. (1972) Hedlund (1984)	Lawrence and Lorsch (1967) Mintzberg (1979)	Jensen and Meckling (1976) Lang and Stulz (1994) Berger and Ofek (1995)
Contributions related to CHQ design	Roles Size Functions	Roles Control Incentives	Location Policies/delegation Internal relations	Policies–coordination Structure	Roles Policies and incentives
Exemplary references	Arrfelt et al. (2015) Chandler (1991) Collis et al. (2007) Feldman (in press) Rumelt et al. (1994)	Foss (1997) Morikawa (2015)	Coeurderoy and Verbeke (2016) Collis et al. (2012) Egelhoff (2010) Meyer and Benito (2016)	Poppo (2003) Sengul and Gimeno (2013)	Scharfstein and Stein (2000) Matolcsy and Wakefield (2017)

^a^ We subsume practice studies of the CHQ by consultancies such as McKinsey & Company, the Boston Consulting Group, and Roland Berger here

However, the twenty-first century is experiencing fundamental changes in the business environment including, among others, new technologies, globalization, and broader societal concerns over business purpose and sustainability that challenge existing knowledge about the CHQ and call for new research into this entity. As the industrial age is on the verge of vanishing to be replaced by the digital age with automation and artificial intelligence increasingly penetrating businesses (Birkinshaw 2018; Iansiti and Lakhani 2020), we have to rethink the role the CHQ plays in companies and the specific functions it performs.

Indeed, the evolving state of modern corporations, where the rise of platforms and ecosystems blurs industry boundaries (Birkinshaw 2018; Cusumano et al. 2019; Davis 2016b), and when public companies are being supplanted by private capital (Jensen 1989) and organizational alternatives, such as cooperatives and mutuals (Davis 2016a; Kolbjørnsrud 2018; Puranam et al. 2014), calls into question the very nature of the CHQ as a value adding and value capturing entity. Furthermore, as companies become more sophisticated and nuanced in the operation of the CHQ with dispersed locations, disaggregated functions and second offices, as well as novel organizational forms that can include virtual headquarters, we need to rethink the size, structure, and location of the CHQ.

The purpose of this article, in keeping with the theme of the *Journal of Organization Design* Special Collection, is to invigorate research into the CHQ believing that the entity offers a window into the nature and functioning of business organizations in the twenty-first century. To do so, we adopt an organization design perspective to provide an integrative lens that links various aspects of CHQ design and builds on contributions from different research streams. Such an integrative perspective allows for deepening and broadening our understanding of the CHQ to offer valuable insights into the conduct of business in the twenty-first century.

We proceed in four steps: first, we introduce a CHQ design perspective as a framing to unify past work across academic disciplines and as a foundation for future research. Second, we discuss fundamental shifts in the business environment that are driving changes in the nature of business organizations in the twenty-first century. Third, we discuss potential implications of those changes for CHQ design and, when appropriate, refer to articles in this Special Collection that describe some of these changes and their impact. Finally, we put forward more general recommendations about further advancement and new research directions, which build on, and extend, contributions from this Special Collection.

## Key elements of CHQ design

We define the CHQ as the central organizational entity, which hosts top executives as well as centralized staff functions that fulfill distinct roles for the overall business entity, which comprised (structurally) separate operating businesses that compete in geographic, product, and customer markets. This is in line with prior definitions (Chandler 1962; Menz et al. 2015) and emphasizes that the multi-business firm is an umbrella term for multinational and multiproduct companies that compete across geographic, product, and customer markets (Chandler 1991). The CHQ is therefore a distinctive feature of diversified business organizations including public corporations, privately owned companies, medium-sized firms, and many family businesses, which account for the majority of economic activity around the world today (McKinsey Global Institute 2013).

Existing knowledge about this entity draws from various academic disciplines and theories including agency, contingency, information processing, and resource-based views (Kunisch et al. 2015; Menz et al. 2015). What unites that research is a common interest in heterogeneities in the CHQ’s design, their determinants and consequences. Asking whether different CHQ designs exist—in terms of roles and functions; size, structure and staffing; location; processes and policies? What are the factors determining those design choices? And what are the performance consequences of those different CHQ designs?

Recognizing that organization design is a contingent phenomenon (Donaldson 2001; Greenwood and Miller 2010; Joseph et al. 2019; Joseph and Gaba 2020), we argue that the elements of CHQ design must be aligned with the firm’s strategy—how the overall business entity creates and captures value across its operating units: whether product divisions and/or country subsidiaries. In turn, that strategy is shaped by an external environment that is rapidly changing in the twenty-first century, for example, technological, demographic, legal, regulatory, and institutional factors affecting the countries and industries in which firms operate.

This perspective suggests those factors and corresponding canonical question whose analysis, in lexicographic order, can guide the design of an effective CHQ. The first question is:

1. How does the overall entity intend to create and appropriate value across individual businesses in the portfolio?

The question echoes Coase’s Nobel Prize winning query, “what is the limit to the scope of the firm?” which challenged us to understand why an extensive array of activities are undertaken within a single organizational hierarchy. In turn, this prompted Rumelt et al. (1994)^2^ to ask, “What is the function, or value added by the headquarters unit in a diversified firm?” as one of the four canonical inquiries in strategic management.

This is the corporate strategy and/or global strategy question, whose answer, drawn from the resource-based view of the firm (Barney 1991; Wernerfelt 1984), depends on the distinctive resources the entity possesses that improve the competitive position of each business in the portfolio (whether product, customer and/or geographic markets) and account for the raison d’être of the CHQ as building a “Corporate Advantage” (Collis 2014; Collis and Montgomery 1998, 2005). The Walt Disney Company, for example, creates value across its numerous businesses through the “multi-platform management of quality branded family entertainment franchises” i.e., making Mickey Mouse, Woody, Darth Vader and the Avengers, available to be exploited in movies, theme parks, hotels, etc. (Collis and Hartman 2020).

Similarly, in IB, Dunning’s eclectic OLI theory (Dunning 1980) argues that to justify the existence of a multinational organization there has to be an *Ownership* advantage based on a distinctive firm specific asset, as well as a *Location* advantage that exploits the resources in a specific geography and an *Internalization* advantage that keeps transactions inside the organizational hierarchy rather than conducted through market exchange. In this regard, Boeing justifies its presence around the world as exploiting global scale economies to cover substantial fixed R&D and manufacturing costs.

Conditional on the answer to the first question is the answer to the second question:

2. What are the critical roles to be played by the CHQ in realizing^3^ the value inherent in the corporate/global strategy by (a) monitoring and controlling discrete units (b) investing in and allocating resources across those units while coordinating their activities and (c) managing external relations?

Originally, Chandler’s (1962) seminal work fueled interest into this question. Later organization studies wrestled with how to balance the differentiation and integration of subunits necessary to realize the value inherent in the multi-business corporation (Lawrence and Lorsch 1967; Lorsch and Allen III 1973)—the classic centralization vs. decentralization dilemma (Galbraith 1973). Relatedly, March (1991) identified the design challenge of structuring an organization to achieve an appropriate balance between exploration and exploitation, and for which others have proposed design solutions (Birkinshaw and Gibson 2004; Raisch and Birkinshaw 2008; Tushman and O'Reilly 1996). The popular book “Strategies and Styles: The Role of the Centre in Managing Diversified Corporations” by Goold and Campbell (1987) further fostered our understanding of this issue.

When those roles have been identified questions three and four must be answered:

3. What is the appropriate size, structure and location for the CHQ functions required to fulfill those roles?
4. Which personnel (top executives and corporate staff), and systems and processes are appropriate for the effective performance of those functions?

In this framework,^4^ a private equity firm controlling a portfolio of diversified operating companies should have a fundamentally different CHQ than a similarly sized public corporation which operates across a tightly related set of businesses (Collis and Anand, in press). The corporate resource(s) which is being leveraged across the business units in each case differs, and so should the roles, size, structure and, possibly even the location, of the CHQ.

## The business environment in the twenty-first century

With these questions in mind, we discuss fundamental changes in the business environment—specifically with respect to technology and globalization, business purpose and governance, and competition—that have altered the organization of business activities in the twenty-first century and so will affect the future design of the CHQ.

### Technology and globalization

As Baum and Haveman (2020, p. 268) argue, “How business is organized is always changing in response to technological, political–economic, or cultural changes. Over the past 50 years, two trends—the rise of digital technologies, including, most recently, the resurgence of artificial intelligence (AI), and the globalization of finance, trade, and production—have fundamentally altered the capabilities and organization of business enterprises and have transformed our ideas about what they should aim to achieve and how organizational theorists should study them.”

#### Technology

With respect to *technology*, just as we saw a profound reconfiguration of organizations in the first machine age during the late nineteenth and early twentieth centuries, so the coming of the digital revolution or second machine age (Brynjolfsson and McAfee 2014) in the twenty-first century^5^ will have a profound impact on a corporation’s scale, scope, and organization (Birkinshaw 2018; Davis 2016b). While the typical large industrial firm in the twentieth century was characterized by vertical integration and clear boundaries that exploited economies of scale and scope, the digital age has brought the rise of horizontally specialized firms with blurred boundaries and virtually unlimited economics of scale (network economies), platforms, and ecosystems (Birkinshaw 2018; Cusumano et al. 2019; McIntyre and Srinivasan 2017). Indeed, the scope of the firm itself is potentially changing as the relative costs of transactions conducted inside a hierarchy as compared with market exchange and contracts, alters, since the more efficient use of information can improve collaboration between individuals who are not collocated, and supports greater modularization of work (Birkinshaw 2018; Davis 2016a).

#### Globalization

While recent events and geopolitical trends might have paused, if not reversed, the post WWII *globalization* trend, reduced physical transportation costs, communications costs, and data storage and analysis costs, have globalized firm supply chains and facilitated geographic expansion (Ghemawat 2017, 2018). The resulting transnational organizations (Bartlett and Ghoshal 1989), global value chains (Benito et al. 2019; Gereffi 2018; Gereffi et al. 2005; Hernández and Pedersen 2017; Strange and Humphrey 2019) and “born global” entrepreneurial ventures (Braunerhjelm and Halldin 2019), pose challenges for the traditional role of the CHQ in multinational firms.

### Business purpose and governance

#### Business purpose

Increasing awareness of the limits of the natural environment have triggered a discussion about the role and purpose of business activities vis-à-vis environmental and social issues (i.e., *sustainability* and *responsibility*), see, for example Henderson (2020) and Anderson (2009). In response, the Business Roundtable (2019) recently redefined the purpose of a corporation as being “for the benefit of all stakeholders—customers, employees, suppliers, communities and shareholders” (see also, Harrison et al. 2020). In a similar vein, Larry Fink, CEO of BlackRock, the world’s largest asset management firm, stated that “To prosper over time, every company must not only deliver financial performance, but also show how it makes a positive contribution to society. … Without a sense of purpose, no company, either public or private, can achieve its full potential. It will ultimately lose the license to operate from key stakeholders”^6^ which prompted Henderson (2020) to observe that for “Fink to suggest that “companies must serve a social purpose” is the rough equivalent of Martin Luther nailing his 95 theses to Wittenberg Castle’s Church door” (p. 10).

While early work in management emphasized that business should contribute to societal goals, after WWII the predominant economic thinking of shareholder value nurtured a single-minded focus on profit maximization (Friedman 1970). However, as business activities have reached levels that increasingly tax natural resources and breach planetary boundaries, an increasing number of scholars, as well as practicing managers, call for change (Howard-Grenville et al. 2019; Nyberg and Wright, in press; Tsui 2020).

Indeed, environmental and social concerns are increasingly being incorporated into the objective of business organizations, reflecting concern over the cumulative effects of their activity on breaching planetary boundaries across a wide range of environmental issues, such as biodiversity and water availability (Rockström et al. 2009; Steffen et al. 2015). Climate change, for example, has become a high priority for many businesses.^7^ These, plausibly, existential threats to the future of human kind have the potential to undermine current business models and create new opportunities with strong support from novel stakeholders, such as NGOs, while also posing challenges for corporate governance as companies face increasing pressure from investors, regulators, activists, and even consumers. For example, “credit rating agencies [now] focus on rising green risks” (Financial Times 2019), while ESG reporting and investing have become increasingly important forces (OECD 2019) to which the CHQ must respond.

#### Governance

There have been important changes in the *governance* of business enterprises as their ownership structure has shifted from the public to the private market, responding to the critique of agency theorists (Jensen and Meckling 1976) who showed how inappropriate managerial incentives could distort corporate decisions and induce behaviors that damaged the interests of shareholders.^8^ Indeed, Jensen’s presciently titled article, “Eclipse of the Public Corporation” (1989), arguing for the superior incentive structure of private ownership has come to pass. Today, the number of public companies in the US is half of what it was in 1998,^9^ while the number of companies owned by private equity exceeded the number of public companies in 2008 (though the value of public companies remains higher) (Wilhelmus and Lee 2018), and 5% of the US GDP is now controlled by private equity (EY 2019).

The alteration in the purpose of the business organization under private ownership—particularly private equity whose legal structure can be considered as a form of CHQ with a tiny corporate office, typically less than 100 employees, responsible for the control of individual portfolio companies—represents a fundamental change in the expectation for how the CHQ is designed to deliver a “corporate advantage” that justifies ownership of multiple businesses and operations in multiple geographies (Collis 2014; Collis and Montgomery 1997, 1998).

### Competition

While in the industrial age of the twentieth century *valuable resources* were primarily physical—oil and land—or intangible supporting physical products, such as the Oreo brand name, or Sharp’s LCD technology, in the twenty-first century the valuable asset can become virtual as data and information become scarce resources.^10^ As an inherently more fungible asset, data should allow firms to extend their scope by entering new businesses. Indeed, the potential is there for companies to pursue radically different business models. Komatsu, for example, no longer just sells earthmoving equipment, but now offers “Smart Construction” as a platform that integrates activities across the entire construction site, from surveying and planning, to the autonomous operation of equipment and coordination of all subcontractors (Collis and Lal 2020). Other novel business models supported by artificial intelligence, machine learning and algorithms that improve productivity (Brynjolfsson and McAfee 2014) include Hitachi Rail offering long-term contracts with performance guarantees for rail service, rather than simply selling the rolling stock (Collis 2020).

More generally, it is the digital space that supports “platform” businesses, like Facebook, Uber, Twitter, and Airbnb, which have revolutionized the economy and created trillions of dollars in value (Cusumano et al. 2019). As these business models sit at the center of an ecosystem of partners, suppliers, and customers the boundary of the firm and hence the role of the CHQ itself come under renewed investigation.

A related change in the twenty-first century, is that any advantage provided by a given set of corporate resources becomes temporary and competition more fluid. No longer is it the case that building a world-scale factory provides advantage for decades [as was the case with Dupont in the titanium dioxide business (Ghemawat 1989)]. Instead, as competitive advantages become fleeting (D'Aveni et al. 2010; D'Aveni and Thomas 2004; McGrath 2013), firms strive to become more agile.^11^ The epitome of this strategy is to possess “dynamic capabilities” (Eisenhardt and Martin 2000; Teece et al. 1997; Winter 2003) that allow a corporation to rapidly shift its business portfolio in pursuit of new opportunities and sources of advantage by “sensing, seizing, and reconfiguring” (Teece 2007). While there are limits to the value of dynamic capabilities (Collis and Anand, in press), firms aspiring to build such capabilities pose real questions for organization design. Simplistically, if continuous exploration for new opportunities becomes more important [particularly in uncertain environments (Birkinshaw et al. 2016; Teece et al. 2016)], there should be more separation of operating units, whether temporally or structurally, which would require an altered role for the CHQ (Nell et al. 2017).

Collectively, these trends affect the context that shapes business strategy and so determines organization design. As the central entity of contemporary organizations, these developments challenge beliefs about the CHQ that have until now relied on evidence from twentieth century industrial enterprises.

## Implications for CHQ design

Ongoing changes in the twenty-first century will perhaps lead to a repeat of what happened to CHQ design in the second half of the nineteenth century. Then the rise of new transportation and communication technologies, such as railroads, telegraphs, and later telephones, contributed to the vertical integration of manufacturing, marketing, and distribution that maximized the operational efficiency of corporations (Baldwin 2019), and first called into being the centrally coordinated departments which formed the CHQ (Chandler 1991). In this section, we discuss how trends in the twenty-first century business environment may affect the design of CHQ, specifically its roles, size, structure, location, personnel, systems, and processes (see Table 2). Among others, we incorporate and refer to the contributions in this Special Collection (see Table 3).

**Table 2:** CHQ in the twentieth versus twenty-first century

Key inquiries	In Menz et al. (2015)	Twentieth century	Twenty-first century
Determinants	External environment	Technology: 1st to 3rd Industrial revolution; 1st Machine age Unlimited natural resources Globalization	Technology: 4th Industrial revolution; 2nd Machine age Planetary boundaries/natural environment Nationalism Additional stakeholders
	Organizational environment	Clear boundaries; hierarchical structures Vertically integrated firms, for example, large industrial firms diminishing economies of scale Public ownership Tangible resources	Blurred boundaries Horizontally specialized firms; network organizations; ecosystems; platforms; network economies Private ownership Intangible resources: data
CHQ Design	CHQ roles and functions	Drive economic outcomes Relatively static “classic” roles	Not purely economic purpose Dynamic roles that allow for transformation and managerial capabilities
	CHQ size and structure	CHQ as single fully integrated entity	Disaggregated and internationally dispersed CHQ CHQ as Hardware *and* Software Increasingly complex CHQ configuration (e.g., multiple HQ layers and blurred boundaries between CHQ and operating units)
	CHQ location and buildings	All CHQ functions at one location	Internationally dispersed CHQ Distance between CHQ and operating units becomes increasingly important Return of the symbolic CHQ (e.g., large campuses of tech firms); back to the cities
	CHQ personnel	Traditional C-suite and functional heads, e.g., strategic planners Predominantly internally focused	Increasing focus on externally facing tasks Diversity of backgrounds and responsibilities
	CHQ systems and processes	Hierarchical and authoritarian	Information and data become central New ways of internal organizing
Outcomes	CHQ outcomes	Efficiency, effectiveness, value added	Efficiency, effectiveness, value added
	Organizational and societal outcomes	Profitability and growth Shareholder value	Profit and purpose Stakeholder value; ESG (environmental, social, and governance) Agility and resilience

**Table 3:** Summary of contributions included in this special issue (in alphabetical order)

Author(s)	Article type	Research streams	CHQ design focus	Key insights for CHQ research (may contain direct quotes)	Phenomena	Methods (used and implied)	Theoretical perspectives
Asakawa (2020)	Research	International business	CHQ roles CHQ structure	Knowledge sharing patterns differ by the type of HQ a subsidiary reports to, i.e., corporate R&D HQ, top management, DHQ, and RHQ	Disaggregation/complexity	Quantitative; survey multi-level	Knowledge-based view (KBV)
Baldwin (2019)	Research	Organization studies	CHQ roles	The technology of a flow process with bottlenecks rewards unified governance, a hierarchical structure, and the use of direct authority, which characterize “modern” industrial corporations with central CHQ	Technology Organizational forms	Historical methods	Theories of organizational design and production
Campbell (2020)	Commentary	Strategic management	Various	Presents several lessons learned related to why CHQ design is important, why it also involves strategy work, what activities are needed at the CHQ level, and the problem of subtracted value	Dynamics and processes	n.a	Practice perspectives
Chasserio and Botte (2020)	Case study	Strategic management	Various	Describes a CHQ transformation that has developed its own participative method with strong reliance on internal resources and an intensive pace of change	Dynamics and processes	Case study temporal and process methods	Practice perspectives
Dellestrand et al. (2020)	Research	International business	CHQ roles	The resource allocation process corresponds to different resource allocation strategies of headquarters (winner-picking and cross-subsidization) and subsidiary behavior (collaboration or competition)	Resource allocation Internal relations	Conceptual	Contingency theory / complementary fit
Foss (2019)	Point of view	Organizational economics	CHQ personnel	A better understanding of the CHQ furthers our understanding of the link between TMT and the rest of the organization, and improves our understanding of the costs and benefits of hierarchical organization	People Internal hierarchy	n.a Multi-level	Organizational economics
Kim and Wu (2019)	Research	Strategic management	CHQ location	CHQ proximity to the firms´ entrepreneurial alliance partners has a positive influence on the innovation performance	(Inter-) Organizational forms	Quantitative; secondary data	KBV Coordination
Laamanen (2019)	Point of view	Strategic management	CHQ design	Developing a better understanding of the dynamics of attention between the different subsidiaries and the increasingly dispersed and disaggregated headquarters activities in MNCs requires a more dynamic view of attention	Disaggregation/complexity	n.a Multi-level Temporal and process methods	Behavioral (ABV)
Lunnan et al. (2019)	Research	International business	CHQ roles	Relationship atmosphere reduces organizing costs, distance increases bargaining costs; centralization and formalization reduce information costs, social integration increases bargaining costs	Coordination, control Internal relations	Quantitative; survey	Transaction cost economics Agency theory
Schmitt et al. (2019)	Point of view	International business	CHQ personnel CHQ processes and systems	CHQ expect to become more powerful and more involved in subunits. CHQ should put emphasis on social interactions for data to be effectively collected and analyzed, for decision-making power to be adequately allocated, and for CHQ involvement to be informed and necessary	Technology; decision-making (biases)	Illustrative; survey	Behavioral/micro-foundations
Sharer (2019)	Commentary	Organization studies	Various	CHQ is a physical place with implications for culture and work processes of the firm and a set of processes and strategies that define how work gets done and employees and leadership groups interact. These elements of hardware and software must be designed and operated together	Various: physical place, culture	n.a Multi-level	Practice perspectives Contingency
Verbeke and Yuan (2020)	Point of view	International business	CHQ personnel	Motivations and abilities determine whether CHQ intervene in subsidiary initiatives and effectiveness of such intervention. Contextual analyses at the MNE level and analyses of individual decision-makers need to be combined when exploring the underlying micro-foundational mechanisms of decisions	Disaggregation/complexity	n.a Multi-level	Behavioral/micro-foundations

### CHQ roles

As the apex of the organization, the CHQ fulfills several internal and external-facing roles. Broadly, the roles of the CHQ can be summarized as: “(1) performing obligatory (public) company functions, also referred to as “minimum CHQ”, including responsibility for oversight of the individual businesses; (2) providing the firm’s operating units with centralized services, such as personnel HR, IT, or media purchasing; and (3) value creation” (Menz et al. 2015: 645). While in our view these generic CHQ roles will continue to exist in the twenty-first century, their qualities and their relative importance may change.

Digitalization can have differential effects on the first, obligatory, CHQ role.^12^ By exploiting information and communication technology (ICT), this role might be pursued more cost effectively, which could reduce the already small size of this function (Kunisch et al. 2012; Morikawa 2015; Young et al. 2000). And, as the ownership structure of corporations’ shifts from the public to the private capital markets, there may be a corresponding reduction in the “public company” functions necessary to deal with external constituencies, such as auditors, ratings agencies and investment analysts, which would, in turn, reduce the workload on the CHQ in its “minimal function” role.

Conversely, with reduced information-processing costs the CHQ is potentially able to take on new tasks, such as data analytics (McKinsey Global Institute 2016), or to become more directly involved in operating units’ decision-making (Bloom et al. 2014). Lower data collection, storage and analysis costs could also create the incentive to incorporate more tasks into the CHQ “core”, even if they are actually value destroying (Campbell 2020; Campbell and Szulanski 2016).

In the twentieth century, the CHQ’s second role of providing shared services was, to a large extent, justified by exploiting scale economies through the provision of central services which internalized activities performed more efficiently within the organizational hierarchy than on a market. Thanks to reduced transaction costs and blurred boundaries between organizations and external parties in the twenty-first century (Birkinshaw 2018), outsourcing of functions has been facilitated, which potentially threatens the advantages of internal shared service functions and so reduces the role and size of the CHQ.

In the twenty-first century, the importance of the third, value creating, role for the CHQ as the raison d’être for the existence of the corporation may change, particularly since the very purpose is being reexamined under the guise of “Stakeholder Capitalism” (Henderson 2020). Indeed, it has almost been an act of faith for researchers in corporate finance (to say nothing of practitioners on Wall Street) to believe in a “conglomerate discount” that denies the existence of value creation in diversified companies (Berger and Ofek 1995; Lang and Stulz 1994; Rajan et al. 2000). While recent research throws some doubt on the belief (Villalonga 2004), finance points to the misallocation of resources by the CHQ across the portfolio as “the dark side of internal capital markets” (Scharfstein and Stein 2000) which can lead to value destruction. Again, others disagree (Hall et al. 2012), but the challenge for the CHQ and its effectiveness in the role of resource allocator to create value is clear from this research stream (see also, Arrfelt et al. 2015; Sengul et al. 2019). As capital markets drive by some of these beliefs, force diversified companies to justify their existence, the pressure to create a corporate advantage, particularly through the use of corporate wide initiatives (Boppel et al. 2013; Collis and Junker 2017; Kunisch et al. 2019a) increases (Collis et al. 2015; Sadun 2018).

In general, there seems to be an increasing importance of external-facing roles for the CHQ, at least in public companies. Birkinshaw et al. (2006) revealed that the CHQ keeps close ties with external stakeholders, in particular global financial markets and shareholders. The increasing importance of sustainability and social responsibility can be expected to further expand the external-facing roles of the CHQ, especially with respect to non-market strategies and external stakeholders, such as NGOs, activist investors, and even consumers.

### CHQ size and structure

Conditional on the roles it must fulfill to achieve “corporate advantage” are the size and internal structure of the CHQ (Menz et al. 2015). There do not appear to be any recent systematic trends in the absolute size of the CHQ (Kunisch et al. 2012; Young and Goold 1993; Young et al. 2000; Zimmermann et al. 2008, 2010), although many companies, like Maersk, have gone through cycles of downsizing and upscaling the number of employees in the CHQ (Collis and Shaffer 2014). Nor have there been consistent changes in the formal organization structure of the corporation. While novel structures may have been experimented with (Gulati et al. 2000, 2012), evidence suggest the continuing predominance of a divisionalized structure in most diversified companies (Sengul 2018).

Nevertheless, the specific functions or activities performed at the CHQ can alter in response to changes in the external environment or the firm’s overall strategy. For example, a survey of 761 of the largest corporations in North America and Europe, revealed that almost a third of companies reported an increase in the number of corporate functions—and fewer than 10% reported a reduction—from 2007 to 2010 (Kunisch et al. 2014). While certain corporate functions such as IT, marketing, HR, and finance exist at most firms, new functions, in areas such as risk management and compliance, are emerging. Digital disruption, for example, both affects the activities of the corporate strategy function at the CHQ which emerged in the 1950s (Menz and Barnbeck 2017; Whittington et al. 2017), and can lead to the creation of a new corporate function at CHQ dedicated to digital transformation (Kunisch et al. 2020; Singh et al. 2020).

However, while the CHQ in the twentieth century enterprise was a relatively clearly identifiable entity, the distinction between CHQ and operating unit activities is less clear in the twenty-first century, as more efficient information processing and better communication technologies allow the CHQ to evolve into a disaggregated entity with central functions housed in different organizational units (e.g., Kunisch et al. 2019b; Nell et al. 2017). Percy Barnevik at ABB was notorious for his 30.30.30 rule for CHQ in the 1980s—30% of its activities should be halted, 30% outsourced to third parties, and 30% pushed down to the operating units—(Bartlett 1993), but his initiatives were, perhaps, merely the precursor to recent changes in the CHQ. Indeed, some authors observe a trend to disaggregation, as large corporate functions, such as finance and HR, are split apart and specific activities moved to the appropriate organizational unit, which is not necessarily the CHQ (Desai 2008; Gospel and Sako 2010).

In line with this more deliberate distribution of CHQ functions among organizational entities, has been a dispersion of those activities across locations in order to capitalize on the optimal site for each unique activity.^13^ The CHQ finance and legal functions, for example, can be distributed across the globe to access the most appropriate institutional, legal, and regulatory framework for their specific task (Birkinshaw et al. 2006; Baaij et al. 2015; Desai 2009). In addition, the disaggregation of CHQ into dual or virtual headquarters (Kunisch et al. 2019b) has obvious implications.

The whole can be thought of as the devolution of many of the previous functions to operating units, subsidiaries, or third parties. As a result, while the change of many corporations from “vertical integrators” to “horizontal specialists” suggests a smaller and simpler CHQ, the corporations’ overall constellation of unit, divisional, group, geographic, regional, and traditional CHQ structures, have become more complex (Nell et al. 2017).

### CHQ location (and buildings)

A key feature of any CHQ in the twentieth century was its, often very visible location and the physical structure and design of the office where its employees sat each day (Menz et al. 2015: 647). While technology companies such as Apple, Facebook, and Amazon still seem to prefer vast corporate campuses on which collocated personnel can easily mingle (The Economist 2016b; c), other corporations are moving the CHQ back from the suburbs to the inner city (The Economist 2016a).

As its size decreases the CHQ becomes increasingly concentrated on a smaller cadre of senior executives, with operational activities transferred to other locations, a downtown location can become more attractive (Collis et al. 2020). The CHQ can be collocated there with important third parties, such as lawyers and advertising agencies; supports easier travel through major airports; and accesses city attractions that appeal to older corporate executives whose children are no longer in the school system. As Buckley and Casson (2020) argue, “In principle, … headquarters activities could be located in different places: the legal headquarters in a low-tax country, the financial headquarters close to a major stock market and the operational headquarters at the centre of transport and communications networks. There is also the question of where the entrepreneur themselves would like to live. There are obvious advantages to co-location, and so locations that meet all four criteria—low tax, good-lifestyle, well-connected financial centres—will attract co-located operations. A distributed headquarters may be useful, however, for specific purposes” (page 9).

While the symbolic nature of the CHQ building and external relations did not receive much scholarly attention (Pirinsky and Wang 2006; van Marrewijk 2009), the choice of location vis-à-vis the firm’s operating units (Kalnins and Lafontaine 2013; Landier et al. 2009), and specifically, the distance between the CHQ and the international subsidiaries (Baaij and Slangen 2013; Dellestrand and Kappen 2012; Parks 1974) has received attention and is believed to influence its effective functioning.

In the twenty-first century, with the emergence of businesses that serve as platforms or important nodes in ecosystems, the location of CHQ in relation to “external” partners becomes an important concern. For example, Kim and Wu (2019) study firm’s strategic alliances and find that proximity of CHQ to alliance partners has a positive effect on innovation performance. More specifically, “a 1000-km decrease in CHQ–partner distance leads to an increase of 28 forward citations for the alliance partner, i.e., a 1% decrease in the distance is associated with a 1.7% increase in innovation performance.”

### CHQ personnel

CHQ personnel include C-suite executives and staff in specialized corporate functions. Research on how the composition of the top management team (TMT) and the Board of Directors affects performance (Finkelstein et al. 2009; Hambrick 2007; Hambrick and Mason 1984) reveals not just that the managerial background and experiences of individual members of the C-suite (e.g., Nath and Mahajan 2011; Waller et al. 1995) as well as the mix of genders, ethnicity, and personal networks among them (e.g., Adams and Funk 2011) has an effect on corporate decisions, but also that the particular job definitions and responsibilities of individuals in the C-suite make a difference.

For example, while the industrial age was characterized by the rise of the Chief Financial Officer in response to the need to acquire financial resources from outside (Zorn 2004), many CHQ’s now contain a Chief Strategy Officer (Breene et al. 2007; Menz and Scheef 2014), Chief Digital Officer (Kunisch et al. 2020), Chief Sustainability Officer (Fu et al. 2019; Kanashiro and Rivera 2019) and Chief HR Officer (Abt and zu Knyphausen-Aufseß 2017; Shi et al. 2018) in response to technological change, changes in task demands, and contemporary sustainability, and social concerns.

In this regard, researchers have also identified increases in the number of direct reports to the CEO (Neilson and Wulf 2012; Wulf 2012) and changes in the skills desired of C-suite members that are attributed to external changes (Svejenova and Luis Alvarez 2017), as the tasks performed by the TMT move towards more integration, external relations, and less direct operating involvement (Fuller et al. 2020).

While there is rich literature on top executives, albeit “imperfectly connected” to organization design theory and CHQ research (Foss 2019), much less is known about the peculiarities of CHQ staff (for a notable exception, see Kleinbaum and Stuart 2011) and changes over time (for a notable exception, see Whittington et al. 2017).

### CHQ systems and processes

Digitalization and globalization have challenged beliefs about the need for and design of integrating mechanisms that tie a corporation together—allowing the CHQ to monitor and control discrete business units, while coordinating their activities and allocating resources among them. An obvious example is the reduction in communication costs on the internet—rather than incurring telecommunication fees, global video conferencing is now essentially free—which increases the potential for interaction between CHQ and operating units, and expands the set of systems and processes that integrate the organization.

Exactly what forms those designs might take—matrix structures, organically evolving networks of loosely contracted parties, artificial intelligence deploying algorithms, restructuring incentive compensation schemes or altering budgeting and capital expenditure policies—is still unclear, but the potential for new approaches and different modes of control (Schafheitle et al. 2020) is readily apparent. Indeed, these changes perhaps shift the balance of the ongoing tension between centralization and decentralization (Bloom et al. 2014; Galbraith 1973).

## Further advancements and new directions

**Fig. 1:**
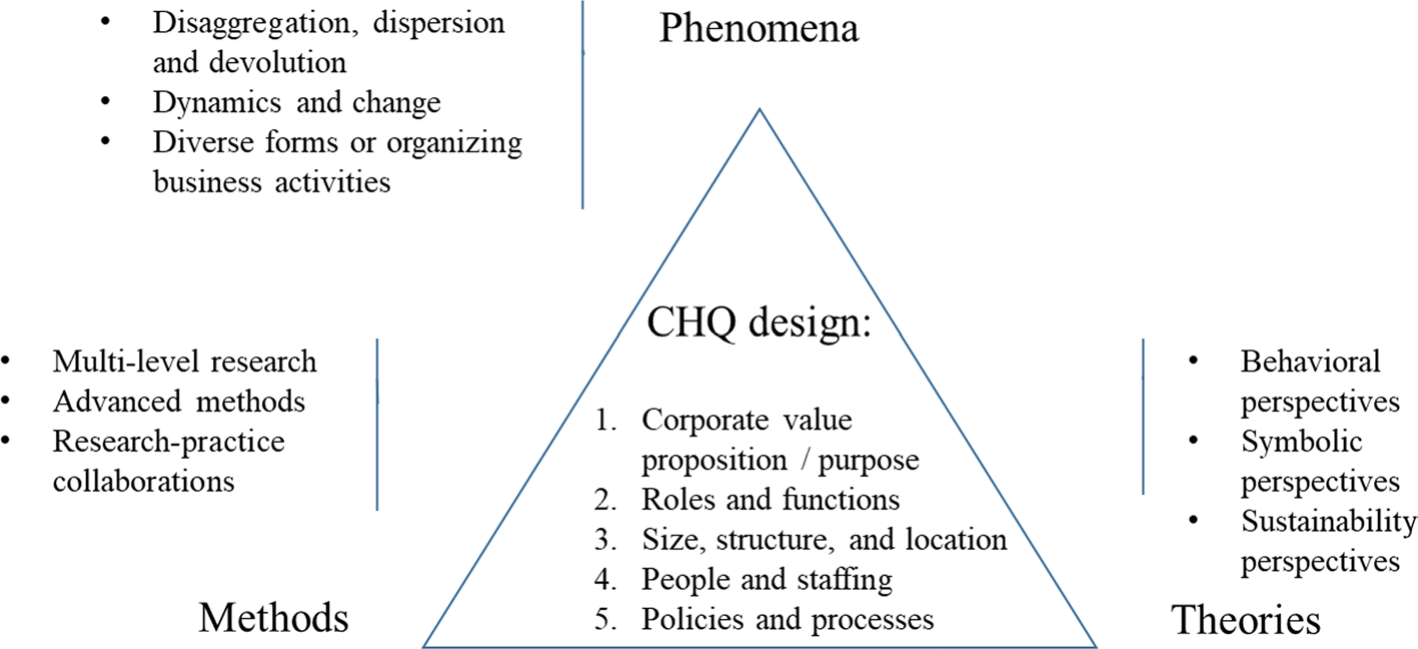
Considerations for future research

We believe that the CHQ offers a unique window into the organization of business activity and, as such, that research into the entity holds the potential to explain heterogeneities in the behavior and performance of traditional, as well as alternative business organizations, in the twenty-first century. While the discussion of the implications for CHQ design in the previous section indicated specific research opportunities, in this section we propose more general recommendations for advancements and new directions. We organize our discussion along the three elements of field research—phenomena, theories/perspectives, and methods (see Fig. 1). As such, we do not strive to suggest specific research projects, although we will refer to exemplary contributions in this Special Collection (see Table 3), but more general considerations to stimulate future research.

### Phenomena

As described in the second and third sections, the business environment has changed since Chandler (1962) first described the phenomenon of the “general office” as the central organizational unit of large diversified public companies. To move forward we suggest three broad considerations for future research.

#### Disaggregation, dispersion, and devolution

In an era of disaggregation, dispersion, and devolution, CHQs are rarely monolithic (Baaij and Slangen 2013; Desai 2009; Kunisch et al. 2019b). Indeed, the predominant view of the CHQ as “a single, identifiable unit in one specific location” (Nell et al. 2017, p. 1121) suffers from the “reductive fallacy” of oversimplification that had, among others, hampered IB research in the past (Nohria and Ghoshal 1994, p. 492). Thus, echoing prior calls (Menz et al. 2015; Nell et al. 2017; Schulte Steinberg and Kunisch 2016), we believe that future research needs to explore the complex nature of the CHQ.

To this end, the most basic question is what exactly defines the CHQ and so what are its boundaries? Collis et al. (2007) and Markides (2002) already noted the challenge to theoretically define the activities performed at the CHQ. In times of disaggregation, dispersion, and devolution this challenge is even more pronounced.

As such, we believe that there are ample opportunities to revisit traditional topics as well as to explore new questions. For example, Asakawa (2020) explores knowledge sharing in MNCs. He finds empirical support for different knowledge sharing patterns when relaxing the assumptions of a single CHQ unit. Schulte Steinberg and Kunisch (2016) argue that accounting for a non-monolithic CHQ is needed to advance understanding of the agency relations inside the firm. This sheds light on role multiplicity at CHQ as well as nested relations inside and outside the firm, and notably relates to the location of CHQ parts. Thus, we need to understand the determinants and implications of firms´ decisions about whether or not to co-locate certain activities. This illustrates the linkages among CHQ design elements, as well as the need for multi-level theorizing (see also, Laamanen 2019; Verbeke and Yuan 2020).

#### Dynamics and change

While much of the existing understanding of the CHQ takes a relatively static perspective, phenomena related to CHQ design are rarely static (Ferlie and Pettigrew 1996; Kunisch et al. 2015). Campbell (2020), for example, maintains that one of his learnings from many years of working with companies is “that CHQ design is always work-in-progress, not only because people change around the top table, but because the way that the CHQ adds value should be continuously evolving as the needs of the businesses change” (p. 5). Studying changes and processual dynamics at the CHQ, therefore, offers unique opportunities to advance our knowledge about the canonical questions into CHQ design.

The in-depth case study of a large-scale CHQ transformation by Chasserio and Botte (2020) offers a good example. The authors set out to explore “how to overcome the inertia of corporate headquarters (CHQ) by examining a CHQ transformation at an industrial multinational that developed its own participative method with strong reliance on internal resources and an intensive pace of change”. They showed how internal change agents orchestrated and implemented this change at the level of CHQ, but with tradeoffs—such as the pace of change and the exclusion of managers from the consultative process—that may have had effects on the implementation of change. Other exemplary questions may include: How does CHQ design change over the organizational life cycle? How does corporate purpose and corporate strategy evolve over time? How does CHQ location evolve over time?

#### Diverse forms of organizing business activities

We believe that future research would benefit from exploring diverse forms of organizing businesses including meta-organizational forms such as networks and ecosystems. Indeed, while public corporations have long been the dominant organizational form for the conduct of business activity, this is not true anymore (Davis 2016a, b). Thus, the scope of enquiry needs to be expanded from traditional public corporations to include other modes of organizing the conduct of business activities. For example, in today’s complex international environment, many business organizations are exposed to a multiplicity of logics, such as public–private, academic–industrial, or social–commercial (Besharov and Smith 2014). Ambos et al. (2020) studied a hybrid multinational organization that pursued multiple goals to explore its inherent tensions and dualities: “While all multinational organizations face the challenge of managing tensions between local integration and global responsiveness, they are increasingly required to pursue additional, often paradoxical, objectives—such as social and commercial goals. However, we know little about how these tensions at the core of the MNC strategy interact.”

Future research should also explore how the CHQ’s scope can or should extend across organizational boundaries since those boundaries are blurring in business networks and ecosystems (Shipilov and Gawer 2020). While the study by Kim and Wu (2019) exemplifies CHQ research in the context of business networks, we still need to learn about the role played by the CHQ in the context of ecosystems and platforms. Such research may build on IB research, which has conceptualized the MNCs as networks, in which the CHQ and the international subsidiaries “are involved in a perpetual bargaining process” (e.g., Andersson et al. 2007) rather than top-down authority in traditional corporate hierarchies. Exemplary questions include: How does the CHQ coordinate value creation and appropriation across firm boundaries? What skills are needed here?

Interesting research opportunities also relate to spatial issues. While prior CHQ research has focused on CHQ location and proximity to subsidiaries, a few studies show that proximity vis-a-vis external stakeholders and proximity to business networks and ecosystems. Kim and Wu (2019), for example, show that CHQ locations are influenced by inter-organizational relationships. Likewise, Faems et al. (2018) show that CHQ proximity matters for knowledge transfer between subsidiaries and unconnected sister alliances.

### Methods

CHQ research has benefitted from a range of research methods including quantitative approaches with large-scale samples using survey or secondary data, as well as qualitative approaches such as in-depth and/or historical case studies, as also illustrated by the contributions in our Special Collection (see Table 3). Along the same lines, we believe that future CHQ research will benefit from utilizing a variety of methods. In particular, we see three considerations as being potentially fruitful.

#### Multi-level research

Many of the CHQ phenomena are multi-level nature. Thus, in line with multi-level theorizing (see also, Laamanen 2019; Verbeke and Yuan 2020), future research should account for multiple levels of analysis. The study by Asakawa (2020) serves as an example. In this study, the author accounts for two levels within the CHQ as well as at subsidiaries. For another example, in their study of the agency relations between CHQ and subsidiaries, Ambos et al. (2019) employ surveys at CHQ and subsidiaries to account for different organizational levels. Similar and even more advanced designs are needed to identify the effects at various levels within (i.e., *intra-CHQ*, *intra-HQ*, *intra-organization*) and across organizational boundaries (i.e., *inter-organization* and *extra-organization*).

#### Advanced methods

We also believe that future research could benefit from novel methods such as simulations (Csaszar 2018), big data (Wenzel and Van Quaquebeke 2018), and machine learning (Choudhury et al. 2019). While CHQ research has been hampered by difficulties to access data due to the political and strategic nature of this entity, methodological advancements may allow for surmounting such challenges. For example, such methods could be used to analyze job descriptions of CHQ personnel to understand changes in CHQ tasks. Interesting research opportunities may relate to using machine learning approaches in analyzing external communication of CHQ such as press releases. For example, Choudhury et al. (2019) use machine learning approaches to facial and text analysis to study CEO’s oral communication styles. For another example, interesting research opportunities may also lie in analyzing social media. In a similar vein, exploiting digital databases that track internal organization linkages, such as virtual meeting recordings and email, can be revealing of informal rather than formal structures and CHQ relationships.

#### Research–practice collaborations

Finally, we believe that there are ample opportunities for research–practice collaborations. Many consulting firms, like McKinsey & Company and the Boston Consulting Group, indicate interest in various CHQ topics in their published studies (Krühler et al. 2012), although their normative recommendations tend to focus on developing archetypes for the role played by the CHQ and how that is determined by the corporate strategy being pursued by the organization. However, benchmarking studies on the size of the CHQ (Kunisch et al. 2012; Young 1993a; Young and Goold 1993; Young et al. 2000; Zimmermann et al. 2008, 2010) reveal that there is demand for such practice driven studies and illustrate the pressure on CEO’s to “right-size” their CHQ (Gilbert-Tersiguel et al. 2019; The Economist 2008, 2014).

Such cooperative research could also explore the practices actually employed at CHQ (Bettis and Blettner 2020; Burgelman et al. 2018). For example, what strategic planning tools are used? How extensive are portfolio management techniques? What is the connection between capital budgeting processes and corporate diversification? Moreover, such cooperative research could help surmount academic difficulties in accessing data that is not readily available from public sources.

### Theories

Much existing knowledge about the CHQ relates to resource-based perspectives and the diversification literature. While there is still a lot to explore in these areas, we believe that understanding the CHQ could benefit from applying novel perspectives, challenging taken-for-granted assumptions, and developing novel concepts and units of analysis.

#### Behavioral perspectives

There are ample opportunities to study aspects of CHQ design from behavioral and micro-foundational perspectives. Such research has grown in many areas of management, strategy and organizational research (Barney and Felin 2013; Felin et al. 2015), but especially in organization design (Puranam 2018; Raveendran et al. 2020) where it builds behavioral foundations to address a variety of issue underlying organization design such as psychological foundations, micro-politics, and human biases.

Future research could explore the socio-psychological aspects of CHQ design to shed light on, for example, the roles of cognition and emotion. In their study of how HQ attention influences subsidiaries in the MNC, Yu et al. (2019) apply “a social psychological lens, proposing that subsidiaries with more HQ attention often deal with higher performance expectation in terms of contributing towards the MNC, and thus, they tend to have a greater participation in the activities that can demonstrate such contribution.” Moreover, we believe that many interesting research questions relate to the micro-politics inside and outside the CHQ. While scholars have repeatedly noted that the CHQ is a highly political entity, our knowledge in this area is rather limited. Conroy et al. (2017, 2018) provide insights into the roles of micro-politics in CHQ–RHQ relationships and the specific skills that subsidiary actors deploy in attempting to influence corporate headquarters in strategically repositioning the subsidiary’s mandate. The authors, “provide a more nuanced, fine-grained understanding of subsidiary influence by illuminating how influence is augmented and enriched through the concomitant effects of subsidiary actors’ social and political skills, whereas political skill involves the ability to leverage social spaces by developing specific influence tactics such as targeting, showcasing and framing.” Another example is the narrative perspective applied by Koveshnikov et al. (2017), which conceptualizes headquarter–subsidiary relations in the MNC as a multi-level discursive struggle between key managers.

Several contributions in this Special Collection cover specific behavioral aspects including CHQ attention (Laamanen 2019), and potential biases facing CHQ executives in decision-making and in operating units (Schmitt et al. 2019; Verbeke and Yuan 2020). Building on these micro-foundations is critical to understanding the roles and functions of the CHQ including its internal relations with operating units and its external relations. Such research also holds potential to connecting CHQ research to parallel research streams on corporate strategy tools, practices and actors.

#### Symbolic perspectives

Much existing research has focused on the (economic) rationale and functional/substantive aspects of CHQ. But, we know comparably little about the symbolic aspects of CHQ. This is surprising as writers have long noted the symbolic dimension of CHQ and even in the twenty-first century we observe many firms erecting edifices for their CHQ (see The Economist (2015) article titled, “Silicon Valley Headquarters: Googledome, or Temple of Doom?”). Thus research into the “tangible/physical HQ” drawing on multiple literatures, especially symbolic perspectives, could be insightful. For example, is the construction of those “palaces” driven by CEO/TMT ego or identity and framing of what the company stands for? As such, we believe that symbolic CHQ offers an interesting phenomenon to advance theories about executive symbolism and symbolic actions (Hambrick and Lovelace 2018; Westphal and Zajac 1994) as well as organizational identity.

#### Sustainability perspectives

Finally, we believe that there is need for research to revisit the raison d’être of the CHQ. Referring back to our initial question into CHQ design about the “corporate value proposition”, we believe that more research is needed to explore what “value” means and “for whom”, especially considering the resurgence of attention to societal issues and businesses impact on the natural environment. Therefore, future research into the roles and functions of CHQ should connect to broader societal topics and shift focus from exploring the CHQ role in driving financial performance to explore alternative metrics, such as ESG (environmental, social, and governance) that are increasingly used by investors.

Directly relating to the canonical questions of CHQ research concerning how value is created and captured, exemplary questions may include: How does the CHQ define the purpose of business organizations? And which stakeholder interests should it represent? What is the CHQ role in building and attaining a sustainable and responsible business? How does the CHQ engage in societal and environmental initiatives? How does CHQ deal with potential tensions such as between profits and purpose (Birkinshaw et al. 2014; Edmans 2020), and short-term and long-term (Brauer 2013; Ortiz-de-Mandojana and Bansal 2016; Ortiz-de-Mandojana et al. 2019)? How does it manage and balance various stakeholder interests? Such research should connect with parallel research on sustainability and responsibility (Bansal and Song 2017), non-market strategy (Mellahi et al. 2016), and firm stakeholders (McGahan 2020).

## Conclusions

The CHQ remains an important managerial and administrative phenomenon, even as it confronts real challenges to its role and nature as a result of dramatic technological and societal changes. It is the continuing evolution of this external environment that alters the design of the CHQ. As such, the CHQ offers a unique opportunity to provide a window into the conduct of business activity in the twenty-first century. We believe that we are now on the cusp of a change in the CHQ as radical as that which saw its original emergence in the late nineteenth/early twentieth century. Exactly what form that change will take remains for practitioners and researchers to inform.

## Data Availability

Not applicable.

## Appendix

See Table

**Table 4:** Acknowledgements of reviewers for this special collection

Name	Affiliation
Bjoern Ambos	University of St. Gallen Switzerland
Michael Beer	Harvard Business School, Harvard University United States
Cyril Bouquet	International Institute for Management Development (IMD) Switzerland
Laura B. Cardinal	Darla Moore School of Business, University of South Carolina United States
Mark Casson	University of Reading United Kingdom
Xavier Castaner	University of Lausanne Switzerland
Asli Colpan	Kyoto University Japan
Kieran Conroy	Queen's Management School, Queen's University Belfast United Kingdom
Dorthe Døjbak Håkonsson	Aarhus University Denmark
Yves L. Doz	INSEAD (Institut Europeen d'Administration des Affaires) France
Michael J. Enright	University of Hong Kong Hong Kong
Michael Goold	Ashridge Strategic Management Centre United Kingdom
Robert Grant	Universita Bocconi Dipartimento di Management e Tecnologia Italy
Anil K. Gupta	Smith School of Business, The University of Maryland United States
Sunil Gupta	Harvard Business School, Harvard University United States
George P. Huber	McCombs School of Business, University of Texas at Austin United States
Aseem Kaul	Carlson School of Management, University of Minnesota United States
Tobias Kretschmer	University of Munich Germany
Markus Kreutzer	EBS Business School, EBS University Germany
Johannes Luger	Copenhagen Business School Denmark
Anoop Madhok	Schulich School of Business, York University United States
Valentina Marano	D'Amore-McKim School of Business, Northeastern University United States
Dana Minbaeva	Copenhagen Business School Denmark
Tom Mom	Rotterdam School of Management (RSM), Erasmus University Rotterdam Netherlands
Børge Obel	Aarhus University Denmark
William Ocasio	Kellogg School of Management, Northwestern University United States
Torben Pedersen	Universita Bocconi Italy
Ulrich Pidun	TU Berlin & The Boston Consulting Group Germany
Mikolaj Jan Piskorski	IMD Business School Switzerland
Arjen Slangen	Katholieke Universiteit (KU) Leuven Belgium
Charles C. Snow	Smeal College of Business, Pennsylvania State University United States
Toby Stuart	Haas School of Business, University of California, Berkeley United States
Steve Tallman	Robins School of Business, University of Richmond United States
Michael Tushman	Harvard Business School, Harvard University United States
Alex James Wilson	Carlson School of Management, University of Minnesota Twin Cities United States
Brian Wu	Ross School of Business, University of Michigan United States
Sangyoon Yi	Korea Advanced Institute of Science and Technology Korea, Republic of

4.

## Footnotes

1 This body of knowledge includes diverse topics such as its roles and functions (e.g., Arrfelt et al. 2015; Chandler 1991; Foss 1997), size and staffing (e.g., Collis et al. 2007, 2012; Morikawa 2015; Young 1993b), structure and subunits (e.g., Farndale et al. 2010; Findikoglu and Lavie 2019; Kunisch et al. 2014; Menz and Barnbeck 2017; Trichterborn et al. 2016), functional disaggregation and geographic dispersion (e.g., Kunisch et al. 2019b; Nell 2017), location (e.g., Birkinshawet al. 2006; Baaij et al. 2004; Coeurderoy and Verbeke 2016; Meyer and Benito 2016), its internal relations with operating businesses (e.g., Ambos et al. 2019; Joseph and Ocasio 2012; Kostova et al. 2016; Sengul and Gimeno 2013), personnel and TMT composition (e.g., Guadalupe et al. 2014; Kleinbaum and Stuart 2014; Kunisch and Bilhuber 2014; Menz 2012; Rajan and Wulf 2006), and outcomes (e.g., Collis et al. 2007; Nell and Ambos 2013).

2 This and the other questions originate from an earlier workshop and special issue in the Strategic Management Journal (see Rumelt et al. 1991; Schendel 1991).

3 In our logic, realizing refers to value capture over time through the ongoing implementation of the corporate strategy.

4 We acknowledge that this framework is normative. Because of this, outcomes of CHQ design, such as cost efficiency, added value, growth, profitability, and ESG (environmental, social, and governance) metrics are implicit in the questions. In other words, aligning CHQ design according to the questions should produce desirable outcomes.

5 Variously referred to as the 1st to 3rd industrial revolutions, and the 4th industrial revolution (Schwab 2017).

6 https://www.blackrock.com/corporate/investor-relations/2018-larry-fink-ceo-letter.

7 For example, the World Economic Forum Global Risks Report (2019) highlights that firms rank environmental risks third among the top five risks by likelihood and fourth by impact.

8 For example, Davis (2016a, b) documents the decline of public corporations and discusses its implications for the business world and society at large.

9 USA Listed Companies found at https://www.theglobaleconomy.com/USA/Listed_companies. Accessed 8/28/2020.

10 We acknowledge that this claim is not undisputed, especially in a longer time horizon, since there is ultimately a finiteness to natural resources.

11 In contrast, McNamara et al. (2003) provide evidence that while hyper-competition increased from the 1970s to the 1980s, it did not increase in the late 1980s to the 1990s. Other research shows that those firms that build resilience perform better in the aftermaths of crises (DesJardine et al. 2019) so that after COVID-19, there seems to be a shift in focus from agility to resilience (e.g., Birkinshaw 2020).

12 We acknowledge Metin Sengul for drawing our attention to the literature about the differential influences of ICT on firm boundaries (e.g., see Rangan and Sengul 2009). This literature suggests that ICT’s effect on firm boundaries depends on the type of ICT. Technologies that reduce coordination costs encourage more outsourcing and smaller firms, whereas technologies that enable more effective internal monitoring lead to more asset ownership and larger firms.

13 Echoing the “historical evidence on how and why headquarters functions have been geographically distributed.” (da Silva Lopes et al. 2018).
